# Moesin Controls Clathrin-Mediated S1PR1 Internalization in T Cells

**DOI:** 10.1371/journal.pone.0082590

**Published:** 2013-12-16

**Authors:** Akira Nomachi, Masanori Yoshinaga, Jaron Liu, Pakorn Kanchanawong, Kiyoshi Tohyama, Dean Thumkeo, Takeshi Watanabe, Shuh Narumiya, Takako Hirata

**Affiliations:** 1 Center for Innovation in Immunoregulative Technology and Therapeutics, Kyoto University Graduate School of Medicine, Kyoto, Japan; 2 Department of Pharmacology, Kyoto University Graduate School of Medicine, Kyoto, Japan; 3 Mechanobiology Institute, Singapore, Singapore; 4 Department of Bioengineering, National University of Singapore, Singapore, Singapore; 5 Department of Fundamental Biosciences, Shiga University of Medical Science, Otsu, Shiga, Japan; University of California, San Francisco, United States of America

## Abstract

The lipid mediator sphingosine 1-phosphate (S1P) regulates a wide range of cellular activities, including vascular maturation, angiogenesis, and immune-cell trafficking. Among the five known receptors for S1P (S1PR1-S1PR5), S1PR1 is a critical regulator of lymphocyte trafficking: its signaling is required for lymphocyte egress from lymphoid organs, while its down-modulation by agonist-induced internalization is a prerequisite for lymphocyte entry into lymphoid organs from the bloodstream. Despite the importance of S1PR1 down-regulation in determining lymphocyte behavior, the molecular mechanism of its internalization in lymphocytes has not been defined. Here we show that agonist-induced S1PR1 internalization in T cells occurs via clathrin-mediated endocytosis and is regulated by moesin, an ezrin-radixin-moesin (ERM) family member. In S1P-stimulated T cells, S1PR1 relocalized within clathrin-coated vesicles (CCVs) and early endosomes, and S1PR1 internalization was blocked when clathrin was pharmacologically inhibited. Stimulating moesin-deficient T cells with S1P failed to induce S1PR1 internalization and CCV formation. Furthermore, treating moesin-deficient mice with FTY720, an S1P receptor agonist known to internalize S1PR1, caused delayed lymphopenia, and lymphocytes isolated from FTY720-treated moesin-deficient mice still responded to S1P ex vivo in chemotaxis assays. These results reveal a novel role for moesin in regulating clathrin-dependent S1PR1 internalization through CCV formation.

## Introduction

Sphingosine 1-phosphate (S1P) is an essential lysophospholipid mediator in cellular responses ranging from vascular maturation and angiogenesis to immune-cell trafficking [1], [2]. The biological activity of S1P is mediated by five G protein-coupled receptors (GPCRs), S1PR1-S1PR5. Among these, S1PR1 is a critical regulator of lymphocyte trafficking. S1P is enriched in blood and lymph but is present at much lower concentrations in lymphoid organs. This S1P gradient, along with its activation of S1PR1 on lymphocytes, is required for lymphocyte egress from lymphoid organs [3], [4]. Upon S1P stimulation, S1PR1 is rapidly phosphorylated at its C-terminal domain by G protein-coupled receptor kinase (GRK) 2, which is involved in receptor desensitization and internalization [5], [6]. In turn, receptor desensitization is required for lymphocytes to overcome their attraction to blood and allow their entry into lymphoid organs [7]. The immunosuppressive drug FTY720 efficiently induces S1PR1 internalization and its subsequent degradation, thus preventing lymphocyte egress from lymphoid organs and inducing profound lymphopenia [8], [9]. Despite the importance of S1PR1 internalization in regulating lymphocyte trafficking, little is known about the molecular mechanism of agonist-induced S1PR1 internalization in immune cells.

Receptor internalization can occur via clathrin-mediated endocytosis or clathrin-independent routes [10]. Clathrin-mediated endocytosis is an important internalization pathway for GPCRs and many other receptors [11]. Clathrin, AP-2, and endocytic accessory proteins assemble at the plasma membrane to form invaginating clathrin-coated pits (CCPs). Some of the CCPs are stabilized, undergo a maturation process, and pinch off to form clathrin-coated vesicles (CCVs). Clathrin-independent endocytosis, which occurs through lipid rafts and a caveolin-mediated pathway, has recently emerged as another important trafficking pathway [12]. Regardless of the internalization pathway, it is generally thought that internalized receptors are trafficked to an early endosome, where they are sorted for degradation, recycling, or both. Rab proteins, which are small GTPases, play a key role in membrane trafficking by recruiting specific effector proteins [13], [14]. Rab5 controls early endocytic transport via its effector proteins, and Rab4 regulates recycling routes. While many GPCRs are thought to be internalized via a clathrin-mediated pathway [15], [16], it is not known if lymphocytes internalize S1PR1 via this pathway.

The highly homologous ezrin-radixin-moesin (ERM) proteins organize the cortical cytoskeleton by linking filamentous actin to the apical membrane of cells. ERM's regulation of the cell cortex has been implicated in such fundamental processes as cell-shape determination, membrane-protein localization, membrane transport, and signal transduction [17], [18]. Importantly, ezrin has been linked to clarthrin-mediated endocytosis of the α1b-adrenergic receptor in transfected HEK-293 cells [19]. In addition, moesin knockdown provokes abnormal clustering of clathrin-coated structures in HeLa cells, implying that moesin is involved in CCV trafficking [20]. However, ERM's role in receptor endocytosis in primary cultured cells, much less immune cells, is largely unknown.

In this study, we investigated the mechanism of S1P-induced S1PR1 internalization in T cells. We found that it occurs via a clathrin-mediated pathway, and that moesin is critical for this process. A moesin deficiency in T cells impaired agonist-induced S1PR1 internalization and CCV formation. Moreover, we showed that FTY720-induced lymphopenia is delayed in moesin-deficient mice, and that T cells from FTY720-treated moesin-deficient mice respond to S1P in chemotaxis assays, indicating that the loss of moesin causes an S1PR1 internalization defect in vivo. These results thus provide new insight into the mechanism of S1PR1 internalization in lymphocytes, which determines lymphocyte trafficking behavior in vivo.

## Results

### S1P induces S1PR1 in T cells to redistribute and colocalize with moesin

**Figure 1:**
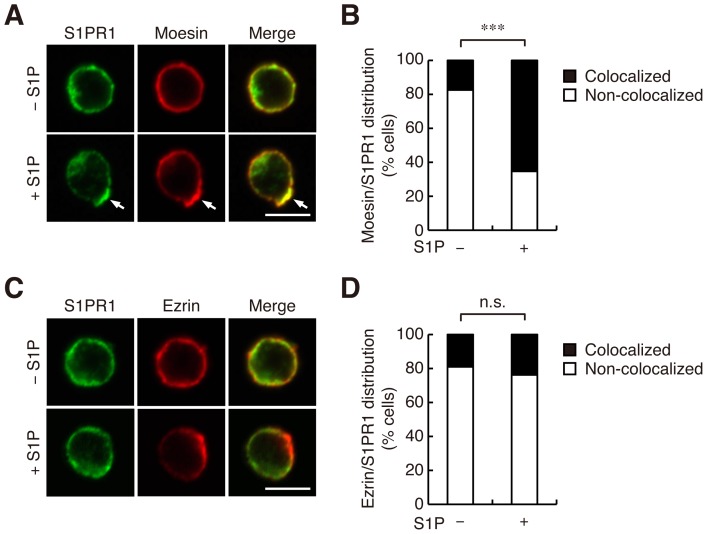
Upon S1P stimulation, S1PR1 redistributes and colocalizes with moesin in T cells. (**A**) S1PR1 and moesin localization in CD4^+^ T cells. Lymph node CD4^+^ T cells from WT mice were stimulated with or without 10 nM S1P for 10 min, fixed, permeabilized, and stained with anti-S1PR1 and anti-moesin. Representative confocal images are shown. Colocalization of S1PR1 and moesin is indicated by arrows. (**B**) Quantification of cells showing S1PR1 and moesin colocalization. The percentage of cells in which S1PR1 and moesin colocalized at cap-like structures was determined. (**C**) S1PR1 and ezrin localization in CD4^+^ T cells. Lymph node CD4^+^ T cells were treated as in (**A**) and stained with anti-S1PR1 and anti-ezrin. Representative confocal images are shown. (**D**) Quantification of cells with S1PR1 and ezrin colocalization. The percentage of cells with colocalized S1PR1 and ezrin at cap-like structures was determined. (**A** and **C**) Scale bars, 5 µm. (**B** and **D**) *n*>60 cells for each group. ***, *P*<0.001; n.s., not significant (Fisher's exact test).

We examined the effect of S1P on S1PR1 intracellular movement in lymphocytes by fluorescence confocal microscopy using a polyclonal antibody that specifically recognizes S1PR1. This anti-S1PR1 antibody stained HEK-293 cells overexpressing S1PR1 (Fig. S1A) and mouse CD4^+^ T cells (Fig. S1B). Preabsorption of this antibody with the antigen peptide abolished the staining (Fig. S1B), and partial suppression of S1PR1 expression in mouse CD4^+^ T cells decreased the staining with this antibody (Fig. S1C and S1D), confirming the specificity. In unstimulated mouse CD4^+^ T cells, S1PR1 localized predominantly to the plasma membrane, where it formed small clusters (Fig. 1A). When cells were stimulated with 10 nM S1P for 10 min, S1PR1 redistributed asymmetrically, with much of it moving to large cap-like structures on the membrane (Fig. 1A).

As ERM proteins have recently been implicated in membrane trafficking [19], [20], we compared the distribution of moesin and ezrin, two ERM members expressed in lymphocytes, with that of S1PR1. In unstimulated CD4^+^ T cells, moesin was evenly distributed at the plasma membrane (Fig. 1A). In cells stimulated with S1P for 10 min, moesin was redistributed asymmetrically, and it colocalized with S1PR1 in cap-like structures in 65% of the cells (Fig. 1A and 1B). Like moesin, ezrin was evenly distributed at the plasma membrane in unstimulated CD4^+^ T cells, and its distribution became asymmetric after S1P stimulation (Fig. 1C). However, the accumulations of S1PR1 and ezrin were mostly distinct, with ezrin and S1PR1 colocalizing in only 24% of the cells after S1P stimulation (Fig. 1C and 1D). Thus, S1P stimulation caused ezrin and moesin to accumulate at distinct membrane domains, and moesin, but not ezrin, colocalized with S1PR1.

### Agonist-induced S1PR1 internalization is impaired in moesin-deficient T cells

**Figure 2:**
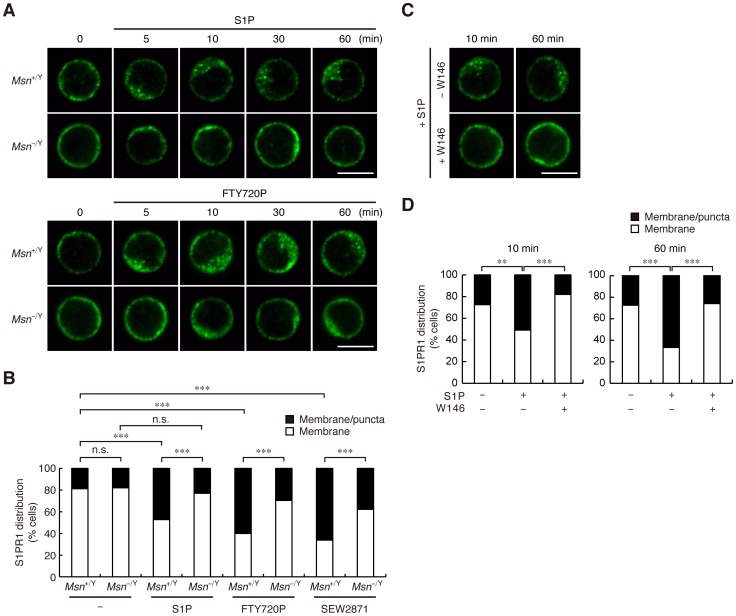
Agonist-induced S1PR1 internalization is impaired in moesin-deficient T cells. (**A**) S1PR1 localization in CD4^+^ T cells. Lymph node CD4^+^ T cells from *Msn* ^+/Y^ and *Msn* ^−/Y^ mice were treated with or without 100 nM S1P or 100 nM FTY720P for the indicated times. Cells were fixed, permeabilized, and stained with anti-S1PR1 antibody. Representative confocal images are shown. Scale bars, 5 µm. (**B**) Quantification of S1PR1 internalization. Lymph node CD4^+^ T cells from *Msn* ^+/Y^ and *Msn* ^−/Y^ mice were incubated with or without 100 nM S1P, 100 nM FTY720P, or 1 µM SEW2871 for 1 h. Cells were stained as in (**A**). Cell percentages with the indicated S1PR1 localization pattern are shown. (**C**) The effect of W146 on S1P-induced S1PR1 relocalization. Lymph node CD4^+^ T cells from WT mice were incubated with or without 1 µM W146 for 30 min and then stimulated with 100 nM S1P for 10 or 60 min. Cells were stained as in (**A**). Representative confocal images are shown. Scale bar, 5 µm. (**D**) Quantification of the effect of W146. Percentages of cells with the indicated distribution patterns of S1PR1 were determined. (**B** and **D**) *n*>50 cells for each group. ***, *P*<0.001; **, *P*<0.01; n.s., not significant (Fisher's exact test).

To study the role of moesin in agonist-induced S1PR1 movement, we compared S1PR1 localization in wild-type (WT; *Msn* ^+/Y^) and moesin-deficient (*Msn* ^−/Y^) CD4^+^ T cells. Stimulating WT cells with 100 nM S1P induced S1PR1 to redistribute as intracellular puncta (Fig. 2A). S1PR1 redistribution was already observed at 5 min after stimulation, and became more apparent after 10 min (Fig. 2A). To quantify the S1PR1 localization, cells were classified according to whether S1PR1 was predominately at the plasma membrane or was intracellular. Prior to S1P stimulation, S1PR1 was predominately localized to the plasma membrane in more than 80% of the WT cells; after S1P stimulation for 1 h, S1PR1 was primarily found intracellularly in 47% of WT cells (Fig. 2A and 2B). The same pattern was seen in unstimulated moesin-deficient cells, but unlike in WT cells, S1P stimulation did not induce S1PR1 to relocalize intracellularly in most moesin-deficient cells at any time points (Fig. 2A). Quantitative analysis showed that S1P stimulation did not significantly increase the percentage of moesin-deficient cells showing the intracellular punctate S1PR1 localization pattern (Fig. 2B).

To examine whether this S1P's action was mediated via S1PR1, we stimulated WT cells with S1P in the presence of W146, an S1PR1-specific antagonist. W146 completely inhibited S1P-induced S1PR1 redistribution (Fig. 2C and 2D), suggesting that S1P induced S1PR1 redistribution through acting on S1PR1. We also examined the effect of stimulation with phosphorylated FTY720 (FTY720P), an agonist for four of the five S1P receptors (S1PR1, S1PR3, S1PR4, and S1PR5), on S1PR1 localization. When WT cells were treated with 100 nM FTY720P, S1PR1 redistributed to intracellular puncta at 5 min after stimulation, and more puncta were observed after 10 min (Fig. 2A). Quantitative analysis showed that the percentage of cells with intracellular S1PR1 localization increased after 1 h stimulation with FTY720P (Fig. 2B). In moesin-deficient cells stimulated with FTY720P, S1PR1 relocalization to intracellular puncta was not observed (Fig. 2A), and the percentage of cells with the intracellular S1PR1 localization pattern was lower than in stimulated WT cells (Fig. 2B). Consistently, when WT cells were treated with 1 µM SEW2871, an S1PR1-specific agonist, for 1 h, the percentage of cells with intracellular S1PR1 localization increased, while it was lower in stimulated moesin-deficient cells than in WT cells (Fig. 2B). In addition, flow cytometric analysis with a monoclonal anti-S1PR1 antibody showed that S1P induced surface S1PR1 down-regulation in WT cells, which was less marked in moesin-deficient cells (Fig. S2). These results suggested that moesin plays a role in agonist-induced S1PR1 internalization. The contribution of moesin to receptor internalization may differ according to receptor type, since CXCL12-induced CXCR4 internalization in moesin-deficient cells did not differ significantly from that in WT cells (Fig. S3A and S3B).

### Internalized S1PR1 localizes to early endosomes

**Figure 3:**
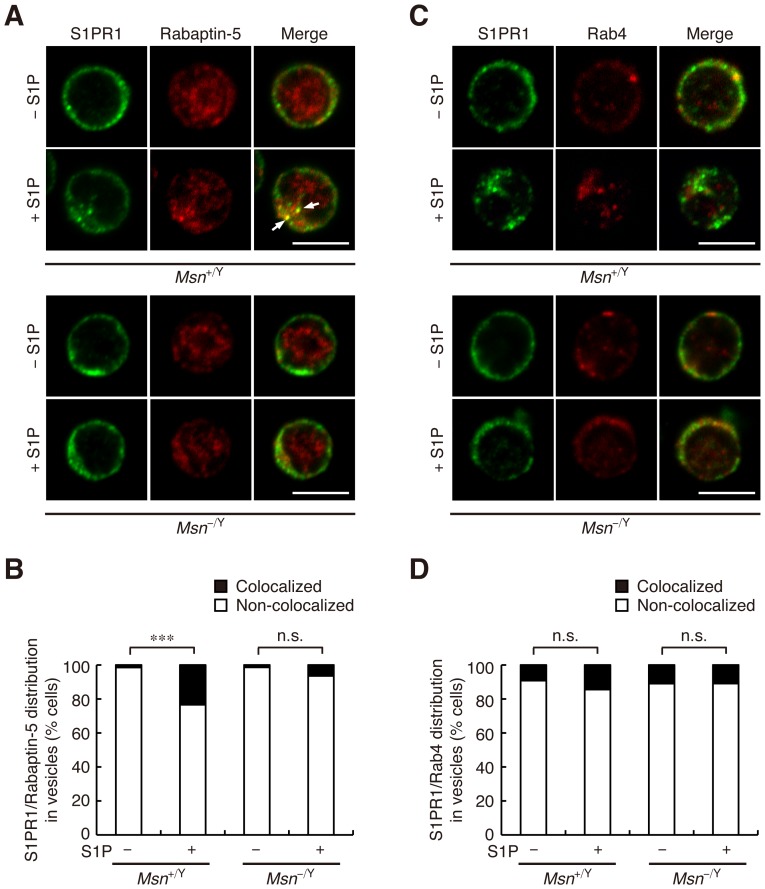
Internalized S1PR1 localizes to early endosomes. (**A**) S1PR1 and Rabaptin-5 localization in CD4^+^ T cells. Lymph node CD4^+^ T cells from *Msn* ^+/Y^ and *Msn* ^−/Y^ mice were incubated with or without 100 nM S1P for 1 h, fixed, permeabilized, and stained with anti-S1PR1 and anti-Rabaptin-5. Representative confocal images are shown. Colocalization of S1PR1 and Rabaptin-5 in vesicles is indicated by arrows. (**B**) Quantification of cells with S1PR1 and Rabaptin-5 colocalized in vesicles. The percentage of cells in which S1PR1 and Rabaptin-5 colocalized in vesicles was determined. (**C**) S1PR1 and Rab4 localization in CD4^+^ T cells. Lymph node CD4^+^ T cells were treated as in (**A**) and stained with anti-S1PR1 and anti-Rab4. Representative confocal images are shown. (**D**) Quantification of cells with colocalized S1PR1 and Rab4. The percentage of cells with S1PR1 colocalized with Rab4 in vesicles was determined. (**A** and **C**) Scale bars, 5 µm. (**B** and **D**) *n*>50 cells for each group. ***, *P*<0.001; n.s., not significant (Fisher's exact test).

Internalized receptors generally traffic to an early endosome, where they are sorted for degradation, recycling, or both. Rab5 controls early endocytic transport through its effector proteins including Rabaptin-5, while Rab4 regulates recycling routes. In unstimulated WT CD4^+^ T cells, Rabaptin-5 was observed predominantly in the cytoplasm, consistent with its localization to early endosomes. Following S1P stimulation for 1 h, the punctate intracelllular S1PR1 staining mostly colocalized with Rabaptin-5 (Fig. 3A). Quantitative analysis showed that internalized S1PR1 and Rabaptin-5 colocalized in vesicles in 23% of the WT cells but in only 6.5% of the moesin-deficient cells (Fig. 3B). In both WT and moesin-deficient cells, internalized S1PR1 and Rab4 did not colocalize in vesicles after S1P stimulation (Fig. 3C and 3D), suggesting that S1PR1 is not predominantly located in recycling endosomes under these stimulation conditions.

**Figure 4:**
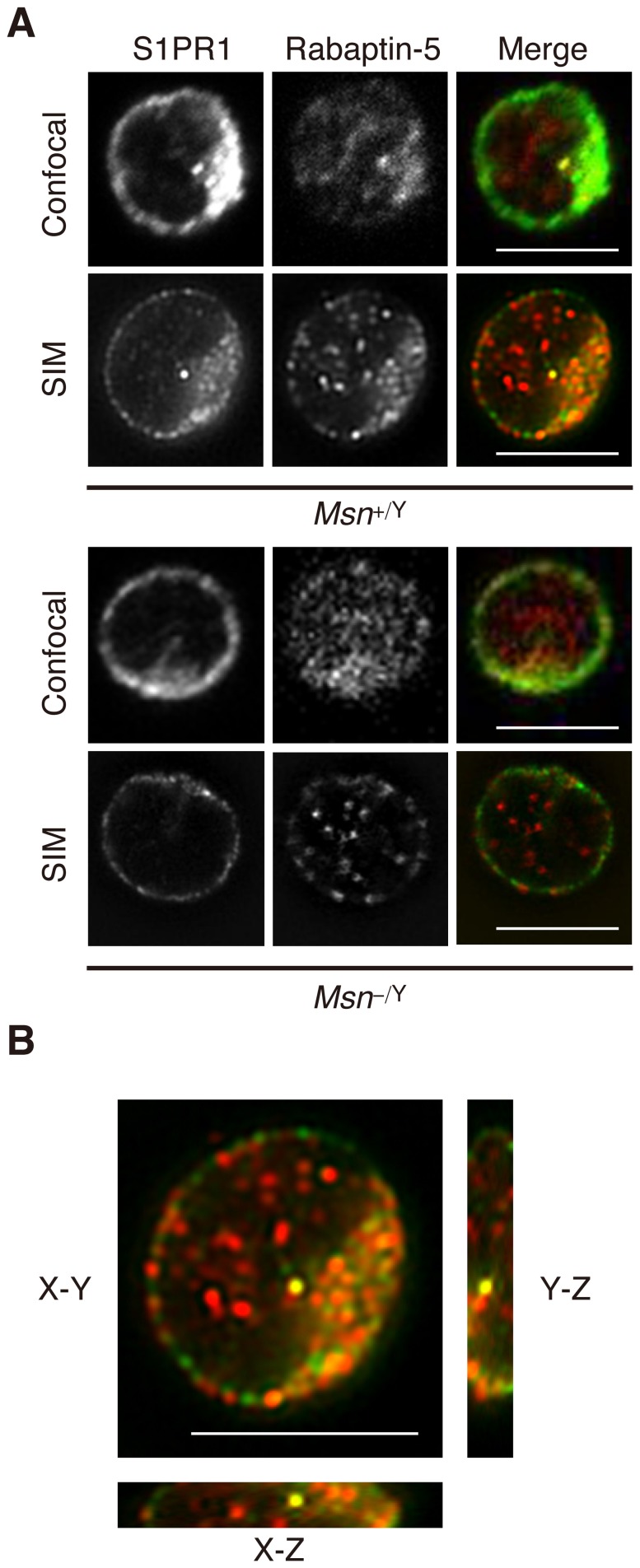
Super-resolution SIM images of S1PR1 and Rabaptin-5 localization in S1P-stimualted CD4^+^ T cells. (**A**) Representative SIM images of S1PR1 and Rabaptin-5 localization in S1P-stimualted CD4^+^ T cells and corresponding confocal images. Lymph node CD4^+^ T cells from *Msn* ^+/Y^ and *Msn* ^−/Y^ mice were incubated with 100 nM S1P for 1 h, fixed, permeabilized, and stained with anti-S1PR1 and anti-Rabaptin-5. Scale bars, 5 µm. (**B**) SIM image with orthogonal side views (XZ, YZ) depicting colocalization of S1PR1 and Rabaptin-5 in S1P-stimulated CD4^+^ T cells from *Msn* ^+/Y^ mice. Scale bar, 5 µm.

To further confirm S1PR1 localization to early endosomes, we visualized these structures by a super-resolution imaging technique, 3-dimensional (3D) structured illumination microscopy (SIM), which provides a lateral (XY) resolution of ∼100–150 nm and an axial (Z) resolution of ∼250 nm (double the resolving power of conventional diffraction-limited microscope in all 3-dimensions) [21]–[23]. In particular, since the size of early endosomes is typically 200–300 nm [24], SIM imaging provides a resolution that is better than or comparable to the size scale of the object, and thus should provide a more unequivocal evidence for colocalization. Based on the super-resolution SIM images, we were able to observe both S1PR1 and Rabaptin-5 as distinct vesicles (Fig. 4A). The comparison for S1PR1 staining between WT and moesin-deficient cells show that the S1PR1 puncta can be observed to be internalized in WT cells, whereas the majority of S1PR1 staining remains mostly on the plasma membrane in moesin-deficient cells. Additionally, we also observed a significant increase in colocalization of S1PR1 and Rabaptin-5 under the S1P-stimulated condition in WT cells (Fig. 4A). To further validate their colocalization in all 3 dimensions, we performed imaging in 3D, with the orthogonal view of the Z-stack SIM images provided in Fig. 4B as well as the animated volume view in Movie S1. Altogether, these results indicate that internalized S1PR1 is sorted mostly to early endosomes.

### S1PR1 internalization requires moesin and occurs via a clathrin-mediated pathway

**Figure 5:**
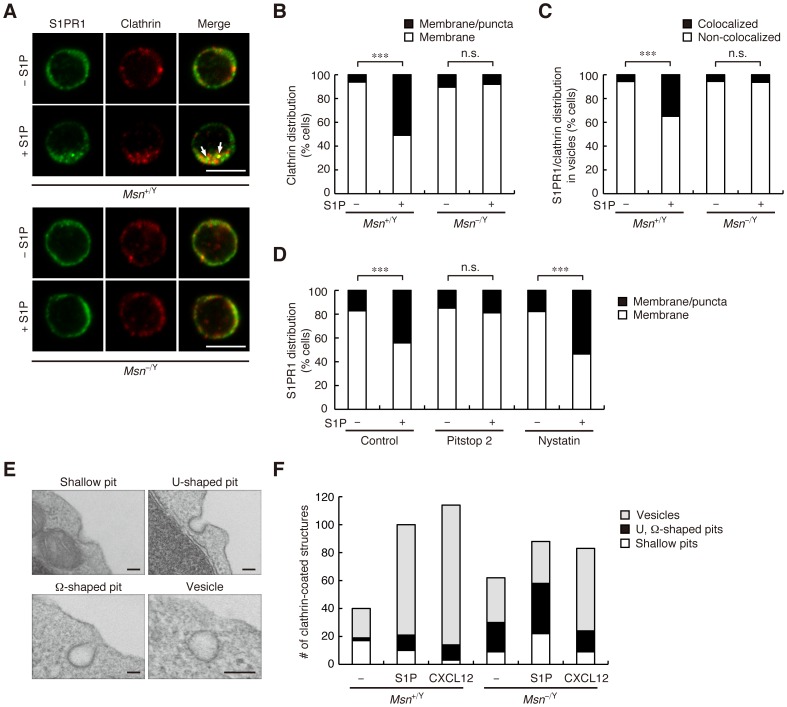
S1PR1 is internalized via a clathrin-dependent pathway. (**A**) S1PR1 and clathrin localization in CD4^+^ T cells. Lymph node CD4^+^ T cells from *Msn* ^+/Y^ and *Msn* ^−/Y^ mice were stimulated with or without 100 nM S1P for 1 h, fixed, permeabilized, and stained with anti-S1PR1 and anti-clathrin heavy chain. Representative confocal images are shown. Scale bars, 5 µm. Colocalization of S1PR1 and clathrin in vesicles is indicated by arrows. (**B**) Quantification of clathrin distribution. The percentages of cells with the indicated clathrin distribution pattern were determined. (**C**) Quantification of S1PR1 and clathrin colocalization. The percentage of cells with S1PR1 and clathrin colocalization in vesicles was determined. (**D**) The effect of pitstop 2 and nystatin on S1PR1 internalization. Lymph node CD4^+^ T cells from WT mice were pretreated with 5 µM pitstop 2 for 30 min or 25 µg/ml nystatin for 1 h, and then stimulated with 100 nM S1P for 1 h in the presence or absence of pitstop 2 or nystatin. Stimulated cells were fixed, permeabilized, and stained with anti-S1PR1. The percentages of cells with the indicated S1PR1 distribution patterns are shown. (**E**) Ultrastructural analysis of clathrin-coated structures. Representative examples of clathrin-coated structures observed at the CD4^+^ T cell plasma membrane are shown. Structures were grouped morphologically as shallow pits, U- or Ω-shaped pits, or vesicles. Scale bars, 100 nm. (**F**) Quantification of clathrin-coated structures observed along the perimeter of CD4^+^ T cells. Lymph node CD4^+^ T cells from *Msn* ^+/Y^ and *Msn* ^−/Y^ mice were stimulated with or without 100 nM S1P or 100 nM CXCL12 for 1 h, fixed, and analyzed by electron microscope. The total number of indicated structures along the perimeter of 25 cells per each group was determined. (**B**–**D**) *n*>50 cells for each group. ***, *P*<0.001; n.s., not significant (Fisher's exact test).

Receptor internalization can occur via clathrin-mediated endocytosis or independent of clathrin. To examine the involvement of the clathrin-mediated pathway in S1PR1 internalization, we analyzed S1PR1 and clathrin localization in unstimulated and S1P-stimualted CD4^+^ T cells. Without stimulation, clathrin localized to the plasma membrane in both WT and moesin-deficient cells (Fig. 5A). Stimulating WT cells with S1P induced clathrin to colocalize with S1PR1 in a punctate intracellular pattern (Fig. 5A); this suggests that S1PR1 localizes to CCVs. This asymmetric punctate staining of clathrin was seen in 51% of S1P-stimulated WT cells, and colocalization of S1PR1 and clathrin was observed in 35% of the cells (Fig. 5B and 5C). In contrast, clathrin remained at the plasma membrane, rather than shifting to the punctate intracellular pattern, in moesin-deficient cells, even after S1P stimulation (Fig. 5A–C). Intriguingly, the punctate and asymmetric clathrin distribution induced by S1P was more marked than that induced by CXCL12 (Fig. S3A and S3C), suggesting that different stimuli induce different clathrin redistribution patterns in T cells.

To confirm that the clathrin-mediated pathway is involved in S1PR1 internalization, we examined S1P-induced S1PR1 internalization in WT cells treated with pitstop 2, which inhibits clathrin-mediated endocytosis by binding to the clathrin terminal domain and thereby blocking endocytic ligand association with this domain [25]. It does not inhibit clathrin-independent routes such as Shiga toxin uptake [25]. S1PR1 internalization was effectively inhibited by pitstop 2, but not by nystatin, which inhibits caveolin-mediated endocytosis (Fig. 5D) [26].

To further clarify moesin's role in clathrin-mediated endocytosis, we used electron microscopy to analyze CCV formation in WT and moesin-deficient CD4^+^ T cells. Clathrin-coated endocytic intermediates were classified according to morphological profile, as vesicles or as shallow, U-shaped, or Ω-shaped pits, as has been described (Fig. 5E) [25]. In unstimulated cells, we found more clathrin-coated structures–particularly U- and Ω-shaped pits–in moesin-deficient cells than in WT cells, suggesting that the moesin deficiency altered constitutive clathrin-mediated endocytosis dynamics. S1P stimulation markedly increased the number of vesicles in WT cells, while in moesin-deficient cells, CCPs were increased but vesicle formation was impaired. Thus, moesin may be involved in the maturation of CCPs or in pinching off vesicles from the plasma membrane. CXCL12 stimulation, however, induced vesicle formation in moesin-deficient cells–albeit at a lower efficiency than in WT cells. Thus, moesin plays a particularly important role in S1P-induced CCV formation. Additionally, transferrin receptor was internalized at a similar efficiency in WT and moesin-deficient cells in response to transferrin stimulation (Fig. S4A and S4B), suggesting that the moesin deficiency does not affect general endocytic events.

### Ezrin is not required for agonist-induced S1PR1 internalization

**Figure 6:**
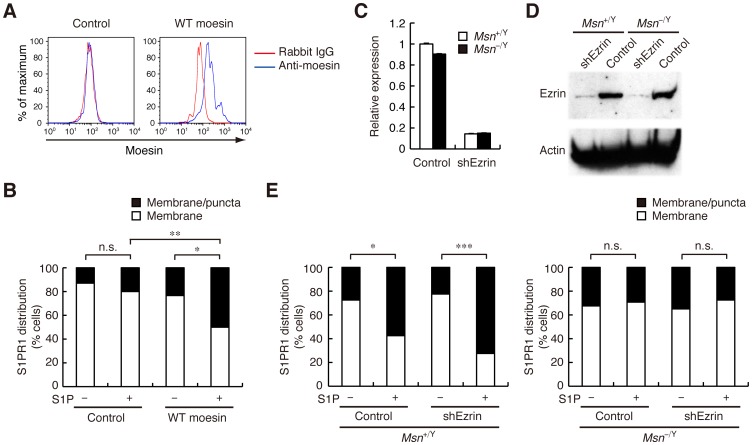
Ezrin is not required for S1PR1 internalization. (**A** and **B**) Reintroduction of moesin into moesin-deficient CD4^+^ T cells. CD4^+^ T cells from *Msn* ^−/Y^ mice were transduced with a control vector or a vector encoding WT moesin; transduced cells were identified by GFP expression. Moesin expression in transduced cells (**A**) and quantification of S1PR1 internalization with or without S1P stimulation (100 nM for 1 h) (**B**) are shown. (**C**–**E**) Ezrin knockdown in CD4^+^ T cells. CD4^+^ T cells from *Msn* ^−/Y^ and *Msn* ^−/Y^ mice were transduced with a control vector or an ezrin shRNA vector (shEzrin). Transduced cells were selected in the presence of puromycin. Knockdown efficiency was confirmed by real-time RT-PCR (**C**) and Western blot analysis (**D**). Quantification of S1PR1 internalization in ezrin-knockdown CD4^+^ T cells with or without S1P stimulation (100 nM for 1 h) (**E**) is shown. (**B** and **E**) *n*>50 cells for each group. ***, *P*<0.001; **, *P*<0.01; *, *P*<0.05; n.s., not significant (Fisher's exact test).

To verify that the moesin deficiency was responsible for the impaired S1PR1 internalization in moesin-deficient T cells, we reintroduced moesin into these cells using a retroviral gene transfer system. Moesin-deficient CD4^+^ T cells were infected with a control retrovirus vector encoding GFP alone (pMXs-IG) or with the same vector, including the coding sequence for WT moesin. Transduced cells were identified by GFP expression and WT moesin expression was confirmed in the moesin-transduced cells (Fig. 6A). S1PR1 internalization was impaired in the moesin-deficient cells infected with the control retrovirus (Fig. 6B), consistent with the impairment observed in untransduced moesin-deficient CD4^+^ T cells. Reintroducing WT moesin, however, rescued S1PR1 internalization (Fig. 6B). The ERM activity is regulated by the phosphorylation of a conserved threonine residue in the C-terminal domain [17]. Reintroducing a phosphomimetic moesin mutant in which the Thr558 was replaced by Asp (T558D) enhanced S1PR1 internalization at the basal level in both WT and moesin-deficient cells in the absence of stimulation (Fig. S5), suggesting that active moesin capable of binding to F-actin is responsible for S1PR1 internalization.

We next investigated ezrin's functional involvement in S1PR1 internalization. Ezrin was distributed at the plasma membrane in moesin-deficient cells, and did not codistribute with S1PR1 after S1P stimulation, much as in WT cells (Fig. S6A and S6B). We infected WT and moesin-deficient CD4^+^ T cells with retrovirus vectors containing ezrin-specific or control shRNA, and confirmed knockdown efficiency by real-time RT-PCR and Western blot analysis (Fig. 6C and 6D). Knocking down ezrin did not affect S1PR1 internalization efficiency in either WT or moesin-deficient cells (Fig. 6E). Taken together, these results indicate that S1P-induced S1PR1 internalization requires moesin but not ezrin.

### FTY720-induced lymphopenia is delayed in moesin-deficient mice

**Figure 7:**
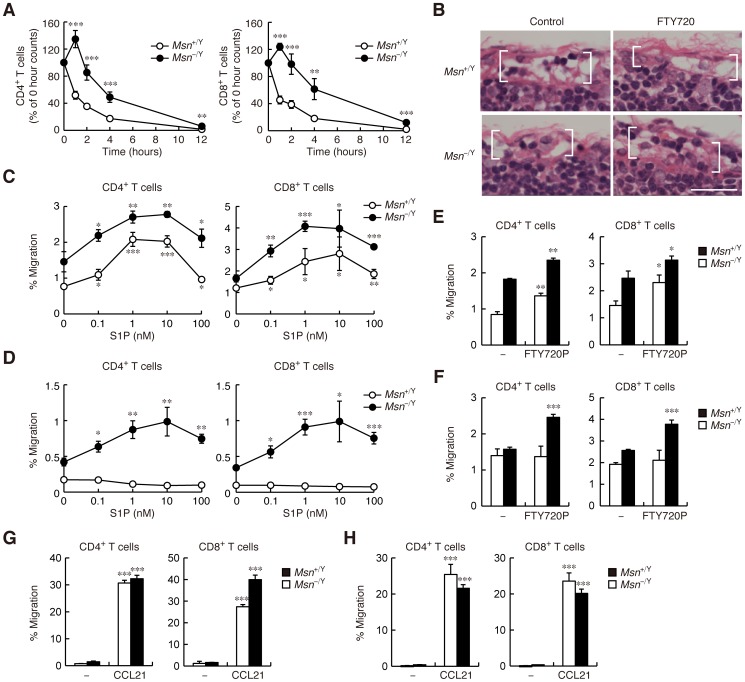
FTY720-induced lymphopenia is delayed in moesin-deficient mice. (**A**) Peripheral blood lymphocyte count after FTY720 treatment. *Msn* ^+/Y^ and *Msn* ^−/Y^ mice were treated with 1 mg/kg FTY720, and peripheral blood samples were collected from mice at the indicated time points. Total leukocytes were counted on a hemocytometer, and the fraction of CD4^+^ and CD8^+^ T cell subsets was determined by flow cytometry. Values are expressed as a percentage of the number of CD4^+^ and CD8^+^ T cells at each time point compared with zero-hour values. Data are representative of three independent experiments, and are presented as means ± s.e.m. ***, *P*<0.001; **, *P*<0.01 versus WT mice (Student's *t* test). (**B**) Histological analysis of lymph node sections from mice treated with PBS or FTY720 for 4 h. Inguinal lymph nodes were harvested and prepared for hematoxylin and eosin staining. Subcapsular sinuses are indicated by brackets. Scale bar, 25 µm. (**C** and **D**) Chemotactic response to S1P in lymphocytes isolated from mice treated with PBS (**C**) or FTY720 (**D**). *Msn* ^+/Y^ and *Msn* ^−/Y^ mice were injected with 1 mg/kg FTY720 or PBS; lymph nodes were isolated 12 h later. (**E** and **F**) Chemotactic response to FTY720P in lymphocytes isolated from mice treated with PBS (**E**) or FTY720 (**F**). (**G** and **H**) Chemotactic response to CCL21 in lymphocytes isolated from mice treated with PBS (**G**) or FTY720 (**H**). (**C**–**H**) Data are representative of three independent experiments and are presented as means ± s.d. of triplicate wells. ***, *P*<0.001; **, *P*<0.01; *, *P*<0.05 versus basal migration (Student's *t* test).

The immunosuppressant FTY720 is phosphorylated in vivo to function as an S1P receptor agonist and to down-regulate S1PR1, and is therefore thought to induce lymphopenia by sequestering lymphocytes in lymphoid organs. FTY720 treatment rapidly decreased both CD4^+^ and CD8^+^ T cells in the blood of WT mice (Fig. 7A). In moesin-deficient mice, the FTY720-induced decrease of blood CD4^+^ and CD8^+^ T cells was significantly slower (Fig. 7A), suggesting a delayed effect. A 4-h FTY720 treatment emptied the subcapsular sinuses of the inguinal lymph nodes in WT mice (Fig. 7B), consistent with lymphocyte sequestration in the lymph node parenchyma. In moesin-deficient mice, however, the subcapsular sinuses still contained some lymphocytes after treatment (Fig. 7B), suggesting that lymphocyte egress through the sinuses was still operative. These results indicate that FTY720 does not cause complete sequestration of lymphocytes in moesin-deficient mice.

We next conducted chemotaxis assays on lymphocytes isolated 12 h after FTY720 or PBS administration. Ex vivo, S1P induced chemotaxis of CD4^+^ and CD8^+^ T cells isolated from PBS-treated mice of both genotypes (Fig. 7C). S1P produced little or no response in CD4^+^ and CD8^+^ T cells isolated from FTY-720-treated WT mice (Fig. 7D), suggesting that FTY720 had already down-regulated S1PR1 in vivo. In contrast, lymphocytes isolated from FTY720-treated moesin-deficient mice still migrated toward S1P to some extent (Fig. 7D). Similar persistent response to FTY720P was observed in CD4^+^ and CD8^+^ T cells from moesin-deficient mice but not in those from WT mice (Fig. 7E and 7F). CCL21 produced a strong response in CD4^+^ and CD8^+^ T cells isolated from both PBS- and FTY-720-treated mice regardless of the genotype (Fig. 7G and 7H). Together, these results suggest that moesin controls S1PR1 down-regulation in vivo.

## Discussion

S1PR1 down-modulation by agonist-induced internalization is an important regulatory mechanism that determines lymphocyte trafficking kinetics in vivo by controlling lymphocyte responsiveness to S1P. Our results show that upon agonist stimulation, T cells internalized S1PR1 through a clathrin-dependent pathway. S1PR1 internalization patterns in moesin-deficient T cells revealed that moesin is critical to this process. This study also offers evidence that moesin controlled the FTY720-induced S1PR1 down-regulation in vivo.

Many GPCRs are regulated through agonist-induced internalization. Clathrin-mediated endocytosis is a key step governing the internalization of many cell-surface receptors, including GPCRs [27]. GPCRs are thought to be rapidly phosphorylated by kinases, such as GRKs, upon agonist stimulation. β-arrestin is then rapidly recruited from the cytosol to the phosphorylated receptor, and the receptor is targeted to CCPs for internalization. Indeed, agonist stimulation of S1PR1 recruits β-arrestin to S1PR1 in Chinese hamster ovary cells engineered to coexpress S1PR1 and β-arrestin [28]. A previous report also speculated that S1PR1 might undergo clathrin-dependent endocytosis in HEK-293 cells overexpressing S1PR1 [29]. In our study, internalized S1PR1 colocalized with clathrin and an early endosome marker, Rabaptin-5, in S1P-stimulated T cells. S1P stimulation induced a strong asymmetric relocalization of clathrin at the membrane and then to intracellular vesicles, while CXCL12 stimulation induced clathrin accumulation largely at the membrane. Additionally, S1PR1 internalization was impaired by inhibiting clathrin-dependent endocytosis pharmacologically. Thus, S1PR1 internalization in T cells occurs via a clathrin-dependent pathway.

Several studies have reported that ERM proteins are involved in endocytosis. Ezrin has been implicated in clathrin-mediated endocytosis of the α1b-adrenergic receptor in transfected HEK-293 cells [19]. Ezrin binds directly to this receptor to regulate receptor recycling. The ERM linker EBP50, also known as NHERF1, is reported to regulate the trafficking of some GPCRs, although the involvement of the ERM proteins themselves has not been demonstrated [30]–[33]. More recently, moesin knockdown was shown to provoke abnormal clathrin-coated structure clustering in HeLa cells [20]. Lymphocytes predominantly express the two ERM members ezrin and moesin, which are regarded as functionally redundant in most situations [34]. We observed that moesin, but not ezrin, colocalized with S1PR1 after S1P stimulation in T cells. We found that the moesin deficiency in T cells substantially impaired agonist-induced S1PR1 internalization and clathrin redistribution to intracellular punctate structures. The use of 3D-SIM confirmed that internalized S1PR1 localizes to early endosomes in T cells. Electron microscopy analysis revealed that, while the moesin deficiency impaired S1P-induced CCV formation, it did not affect CCP assembly. These results suggested that moesin is dispensable for CCP initiation but is required for pit maturation or vesicle scission.

We observed that the moesin deficiency delayed FTY720-induced lymphopenia in vivo. Sphingosine kinase 2 converts FTY720 into its active phosphate-ester form (FTY720P), which is a potent and nonselective S1P receptor agonist [35], [36]. FTY720P blocks lymphocyte egress from lymphoid organs and induces profound lymphopenia, primarily by inducing lymphocytes to internalize and degrade S1PR1 [8], [9], [37]. A transient increase in peripheral blood lymphocyte numbers in moesin-deficient mice suggests that the FTY720's agonistic activity is prolonged due to impaired down-regulation of S1PR1. Delayed lymphopenia has been reported in mice with a knock-in mutation of the S1PR1 serine-rich C-terminal motif [38]. Agonist-induced S1PR1 internalization was delayed in T cells expressing the mutant S1PR1, suggesting that defective S1PR1 internalization leads to delayed lymphopenia. Chemotaxis assays showed that T cells isolated from FTY720-treated moesin-deficient mice still responded to S1P ex vivo, indicating that moesin plays a role in S1PR1 internalization in vivo.

In conclusion, our study provides evidence that moesin plays an important role in clathrin-mediated S1PR1 internalization in T cells. Clathrin-mediated endocytosis regulates not only receptor internalization, but also a variety of cellular physiological processes that include pathogen entry and synaptic transmission. The identification of moesin as a constituent of the complex molecular machinery associated with the clathrin-dependent pathway will help us to understand and control S1PR1 membrane trafficking, which is critical for determining lymphocyte migratory behaviors and other processes involving clathrin-mediated endocytosis.

## Materials and Methods

### Reagents and antibodies

FTY720, SEW2871, FTY720P, and W146 were purchased from Cayman Chemical (Ann Arbor, MI), pitstop 2 from Abcam (Tokyo, Japan), S1P and nystatin from Sigma (St. Louis, MO), and mouse CXCL12 and CCL21 from R&D Systems (Minneapolis, MN). Rat monoclonal antibodies to moesin or ezrin were provided by S. Tsukita (Osaka University, Osaka, Japan). Rabbit anti-moesin antibody (Q480) and rat anti-ezrin monoclonal antibody (M11) were purchased from Cell Signaling Technology (Danvers, MA) and Sanko-Junyaku (Tokyo, Japan), respectively. Anti-S1PR1 antibody (ab11424), generated by immunizing rabbits with a synthetic peptide (SHPQKDDGDNPETI) corresponding to amino acids 359–372 of mouse S1PR1, was purchased from Abcam. Anti-β-actin was purchased from Sigma. Anti-clathrin heavy chain, anti-Rab4, anti-Rabaptin-5, and biotinylated anti-CXCR4 were purchased from BD Biosciences (San Jose, CA). HRP-conjugated anti-rat IgG was purchased from American Qualex (San Clemente, CA), HRP-conjugated anti-mouse IgG from Millipore (Temecula, CA), Alexa Fluor 488-conjugated anti-rabbit IgG, Alexa Fluor 594-conjugated anti-rat IgG and anti-mouse IgG, and Alexa Fluor 488-conjugated streptavidin from Invitrogen (Carlsbad, CA), PE-labeled goat (Fab')_2_ anti-rabbit IgG from Beckman Coulter (Fullerton, CA), and control rabbit IgG from Millipore.

### Mice

Moesin-deficient mice were provided by S. Tsukita and backcrossed more than 10 generations on the C57BL/6 (B6) genetic background [39], [40]. Male *Msn* ^−/Y^ mice and littermate WT *Msn* ^+/Y^ mice were used for all experiments. B6 mice were purchased from Japan SLC (Hamamatsu, Japan) and CLEA Japan (Tokyo, Japan). For FTY720 treatment, *Msn* ^−/Y^ mice and littermate *Msn* ^+/Y^ mice received 1 mg/kg FTY720 or PBS by intraperitoneal injection. The mice were housed at the Institute of Experimental Animal Sciences at the Kyoto University Graduate School of Medicine. All mice used were sacrificed by cervical dislocation under deep anesthesia with sevoflurane (Maruishi Pharmaceutical, Osaka, Japan).

### Ethics statement

This study was carried out in strict accordance with the recommendations in the Guide for the Care and Use of Laboratory Animals of the National Institutes of Health. All procedures were approved by the Animal Research Committee of the Kyoto University Graduate School of Medicine.

### T cell isolation

CD4^+^ T cells were isolated from the spleen or peripheral lymph nodes using a CD4^+^ T Cell Isolation Kit (Miltenyi Biotec, Bergisch Gladbach, Germany). Isolated T cells were starved in RPMI medium containing 10 mM HEPES and 0.1% fatty acid-free BSA for 3 h before use.

### Retroviral vector construction

Full-length mouse moesin cDNA, amplified from splenic CD4^+^ T-cell cDNA, was inserted into a pMXs-IG vector at the *Eco*RI site (vector provided by T. Kitamura of the University of Tokyo, Tokyo, Japan). This vector's long terminal repeats drive moesin and enhanced GFP expression via a bicistronic mRNA with an internal ribosomal entry site element.

To construct vectors expressing shRNA, we used an RNAi Target Sequence Selector (Clontech, Mountain View, CA) to select a targeting sequence of mouse ezrin (5′-GGCCAAGTTCGGAGATTAT-3′). An oligonucleotide pair was designed using shRNA Sequence Designer (Clontech). Annealed oligonucleotides for ezrin shRNA or for negative control shRNA (Clontech) were inserted into the RNAi-Ready pSIREN-RetroQ vectors (Clontech) between the *Bam*HI and *Eco*RI sites.

### Retroviral infections

Improved retrovirus packaging (PLAT-E) cells were transfected with one of the retrovital vector constructs described above, using FuGENE HD Tranfection Reagent (Roche, Indianapolis, IN). The medium was replaced 24 h after the transfection, and the cells were cultured for an additional 48 h. Viruses were harvested in the PLAT-E supernatant, which was either used for infection immediately, or concentrated using a Virus Precipitation Kit (BioVision, Milpitas, CA) and stored until use.

For T-cell spinoculation, purified CD4^+^ T cells were stimulated with plate-immobilized anti-CD3ε (10 µg/ml; 145-2C11; BioLegend, San Diego, CA) plus anti-CD28 (10 µg/ml; 37.51; BioLegend) in the presence of IL-2 (10 ng/ml; R&D Systems) for 18–24 h, harvested, and added along with virus-containing supernatant to a 6-well plate coated with 15 µg/ml recombinant fibronectin fragment CH296 (RetroNectin; Takara, Otsu, Japan). The plates were centrifuged at 2,000× g for 1 h. The cells were then incubated for 24 h, reinfected by spinoculation, and cultured for an additional 3 days in the presence of IL-2 (20 ng/ml).

For RNAi experiments, transduced cells were selected by adding puromycin (4 µg/ml) 2 days after the first spinoculation and incubating them for 3 days. Dead cells were removed by density gradient separation using Ficoll-Paque PLUS (GE Healthcare, Buckinghamshire, UK). The transduced cells were restimulated for 18–24 h with plate-immobilized anti-CD3ε plus anti-CD28, and then cultured for 5 days. IL-7 (10 ng/ml; R&D Systems) was added to the medium during the last 3 days of culture.

### Flow cytometry

For intracellular staining of moesin, cells were fixed and permeabilized using the BD Cytofix/Cytoperm Fixation/Permeabilization Kit, and labeled with a rabbit anti-moesin antibody or control rabbit IgG followed by PE-labeled goat (Fab')_2_ anti-rabbit IgG. Data were acquired on a FACSCalibur (BD Biosciences) and analyzed using FlowJo (Tree Star, Inc., Ashland, OR).

### Quantitative RT-PCR

Total RNA was isolated from T cells using the RNeasy Micro Kit (Qiagen, Valencia, CA). The High Capacity RNA-to-cDNA Kit (Applied Biosystems, Carlsbad, CA) was used for RT. PCR was performed in a final volume of 20 µl containing the cDNA, 1× LightCycler 480 Probes Master (Roche), and 1× Taqman Gene Expression Assay for ezrin (*Ezr*; Mm0044761_m1) or cyclophilin B (*Ppib*; Mm00478295_m1) (all from Applied Biosystems) using a LightCycler 480 (Roche).

### Western blot analysis

Cells were lysed in SDS sample buffer, incubated at 95°C for 5 min, cooled on ice, and centrifuged to obtain supernatant. The samples were electrophoresed on a 5–20% SuperSep Ace (Wako, Osaka, Japan) and blotted to a Clearblot P membrane (Atto, Tokyo, Japan). The membranes were probed with a rat anti-ezrin antibody followed by HRP-conjugated anti-rat IgG. The membrane was also stripped and probed with anti-β-actin.

### Immunofluorescence

Starved CD4^+^ T cells were added to 6-well tissue culture plates containing glass coverslips coated with 10 µg/ml fibronectin. Cells were allowed to settle for 10 min at 37°C and then stimulated with the indicated concentrations of S1P, CXCL12, or vehicle for the indicated times. Cells were fixed in 4% paraformaldehyde in a phosphate buffer for 10 min and permeabilized with 0.5% Triton-X in PBS for 3 min. Fixed and permeabilized cells were blocked with 1% BSA in PBS for 1 h. The cells were stained with the appropriate primary antibodies at 4°C overnight, washed, and incubated with labeled secondary reagents for 45 min at room temperature. The cells were finally washed and mounted with Fluoromount G. Fluorescent images were obtained with a laser scanning confocal imaging system (LSM710; Carl Zeiss Inc., Thornwood, NY) using an oil immersion objective lens (Plan-Apochromat, 63×1.4 numerical aperture) at 512×512 pixels.

To quantify the distribution of S1PR1, the appearance of the intracellular localization of S1PR1 was classified into two types. The first type showed clear localization at the cell surface, with no puncta in the cytosol. The second type showed localization to both the cell surface and puncta in the cytosol. Puncta were defined as distinct, spatially limited fluorescence signals, which had intensity at least five times above background and were larger than 4 pixels. Clathrin distribution to membrane and puncta was quantified similarly. To quantify the colocalization of S1PR1 with Rabaptin-5, Rab4, or clathrin in vesicles, cells were classified according to whether the staining of the two molecules overlapped in puncta in the cytosol. For quantification of colocalization of moesin or ezrin with S1PR1, cells were classified according to whether the two molecules colocalized in cap-like structures. More than 50 cells were evaluated in each experiment in a blind fashion by two independent observers.

### SIM

SIM imaging was performed using a Nikon N-SIM microscope system consisting of a Nikon Ti-E motorized inverted microscope with perfect focus system, a motorized stage with XY linear encoder, a Z piezoelectric stage, 488 and 561 nm 50 mW Coherent Sapphire laser, Chroma ET GFP and ET mCherry filter sets, and Andor IXon3 DU897 EMCCD camera. The objective used is CFI Apochromat TIRF 100× (NA 1.49, oil immersion). The patterned excitation is created by Nikon 100 Ex V-R grating.

For acquiring a 3D-SIM raw data set, 21 Z-stacks with a step size of 0.05 µm were collected with 5 different phase-shifts and 3 different rotation angles of the striped illumination pattern, thus summing up to 15 acquisitions (5 phases times 3 angles) per plane and color channel. To avoid bleed through in multicolor applications, the fluorescence channels are typically collected sequentially for every phase grating position and at every z-position. Image reconstruction was performed using Nikon's NIS-Elements AR software v4.11 with an algorithm based on Gustafsson et al. [21] (reconstruction parameter settings as follows: structured illumination contrast 2, apodization filter parameter 0.7, width of 3D-SIM filter 0.1).

### Electron microscopy analysis

Starved CD4^+^ T cells were stimulated with 100 nM S1P, 100 nM CXCL12, or vehicle for 1 h. Cells were fixed with 2% glutaraldehyde in PBS, embedded in epoxy resin, and sectioned. Micrographs were taken along the cell perimeter at x25,000, x30,000, and x40,000. Images were acquired using Transmission Electron Microscope (H-7650, Hitachi, Tokyo, Japan). Images were combined to reconstruct cell perimeters, and the number of clathrin-coated intermediates per 25 cells was analyzed for each condition.

### Peripheral blood counts

Blood was collected from the tail vein at the time points indicated in the figures after FTY720 injection. The blood was diluted in Türk stain solution (Nacalai Tesque, Kyoto, Japan), and total leukocyte counts were determined using a hemocytometer. Flow cytometry of whole blood was performed to determine the fractions of each subset as described previously [41].

### Histological analysis

For histological analysis, inguinal lymph nodes were harvested from moesin-deficient and WT mice 4 h after treatment with PBS (control) or FTY720 (1 mg/kg). Lymph nodes were fixed in 10% neutral buffered formalin, sectioned at 3 µm, and stained with hematoxylin and eosin.

### Chemotaxis assay

Lymphocytes were isolated by mechanical disruption between the frosted ends of glass slides, filtered through 70-µm nylon mesh, and further purified by density centrifugation through Lympholyte-M (Cedarlane, Hornby, Canada). Lymphocytes were then loaded into 5-µm Transwells (Corning Costar Corp., Corning, NY) (1×10^6^ cells/well), allowed to migrate toward S1P, FTY720P, or CCL21 at indicated concentrations for 4 h, then harvested and counted on a hemocytometer. Subsets were determined by flow cytometry. Transwell assays were performed in triplicate for each concentration.

### Statistics

Results are expressed as means ± s.e.m. in Fig. 6A and means ± s.d. in Fig. 6C-F. Statistical analysis was performed using Student's *t* test. Results were considered significant for *P*<0.05. For quantification of confocal images, statistical analysis was performed using Fisher's exact test. All experiments were performed at least three times.

## Supporting Information

Figure S1 **Characterization of the anti-S1PR1 antibody used in this study.** (**A**) Staining of HEK-293 cells with the anti-S1PR1 antibody. Full-length mouse S1PR1 cDNA, amplified from splenic CD4^+^ T cell cDNA, was inserted into a pFLAG-CMV4 vector (Sigma) between the *Hind*III and *Xba*I sites. HEK-293 cells were transfected with a pFLAG-CMV4 vector encoding mouse S1PR1 (FLAG-S1PR1) or a control vector. The transfected cells were fixed, permeabilized, and stained with the anti-S1PR1 (5 µg/ml; ab11424) or control rabbit IgG followed by Alexa Fluor 488-conjugated anti-rabbit IgG. The cells were costained with anti-FLAG (M2; Sigma) followed by Alexa Fluor 594-conjugated anti-mouse IgG. The anti-S1PR1 antibody stained only cells transfected with FLAG-S1PR1. Representative confocal images are shown. Scale bar, 20 µm. (**B**) Staining of CD4^+^ T cells with the anti-S1PR1 antibody. Mouse CD4^+^ T cells were fixed, permeabilized, and stained with the anti-S1PR1 (5 µg/ml; ab11424) or control rabbit IgG followed by Alexa Fluor 488-conjugated anti-rabbit IgG. The cells were also stained with the anti-S1PR1 pre-absorbed with the antigen peptide (ab39763; Abcam) at a molar ratio of 1∶20 overnight. Representative confocal images are shown. Scale bars, 5 µm. (**C** and **D**) S1PR1 mRNA expression (**C**) and quantification of staining with the anti-S1PR1 antibody (**D**) after partial suppression of S1PR1 in CD4^+^ T cells. Mouse CD4^+^ T cells were transfected with siRNA for S1PR1 (Invitrogen) with Neon Transfection System (Invitrogen). After 72 h, total RNA was isolated and subjected to quantitative RT-PCR for S1PR1. Transfected cells were stained with the anti-S1PR1 (5 µg/ml; ab11424) and quantification of fluorescence intensity and area in each cell was performed using ImageJ software (http://rsbweb.nih.gov/ij). ***, *P*<0.001; *, *P*<0.05 (Student's *t* test).
(TIF)

Figure S2 **S1P induces cell-surface S1PR1 down-regulation in CD4^+^ T cells.** Lymphocytes from *Msn* ^+/Y^ and *Msn* ^−/Y^ mice were stimulated with or without 100 nM S1P for 1 h. The cells were stained with a rat monoclonal antibody to mouse S1PR1 (40 µg/ml; MAB7089; R&D Systems) at 40 µg/ml or control rat IgG2a for 30 min at room temperature, and then stained with biotinylated anti-rat IgG (Jackson ImmunoResearch, West Grove, PA) followed by allophycocyanin-conjugated streptavidin (BD Biosciences). Lastly, the cells were surface-stained to enable identification of CD4^+^ T cell subsets. Data were acquired on a LSRFortessa (BD Biosciences) and analyzed using FlowJo. Rat IgG2a staining of S1P-stimulated cells (not shown) largely overlapped to that of unstimulated cells.
(TIF)

Figure S3 **CXCL12-induced CXCR4 localization in CD4^+^ T cells.** (**A**) CXCR4 and clathrin localization in CD4^+^ T cells. Lymph node CD4^+^ T cells from *Msn* ^+/Y^ and *Msn* ^−/Y^ mice were incubated with or without 100 nM CXCL12 for 1 h, fixed, permeabilized, and stained with anti-CXCR4 and anti-clathrin heavy chain. Representative confocal images are shown. Scale bars, 5 µm. (**B**) Quantification of CXCR4 internalization. Percentages of cells with the indicated distribution patterns of CXCR4 were determined. (**C**) Quantification of clathrin distribution. The percentages of cells showing the indicated distribution pattern of clathrin were determined. (**B** and **C**) *n*>50 cells for each group. ***, *P*<0.001; n.s., not significant (Fisher's exact test).
(TIF)

Figure S4 **Transferrin-induced transferrin receptor internalization in CD4^+^ T cells.** (**A**) Transferrin receptor (TfR) localization in CD4^+^ T cells. CD4^+^ T cells from *Msn* ^+/Y^ and *Msn* ^−/Y^ mice were incubated with or without 20 µg/ml transferrin (Invitrogen) for 10 min, fixed, permeabilized, and stained with an anti-TfR antibody (Abcam). Representative confocal images are shown. Scale bars, 5 µm. (**B**) Quantification of TfR internalization. Percentages of cells with the indicated distribution patterns of TfR were determined. *n*>50 cells for each group. ***, *P*<0.001 (Fisher's exact test).
(TIF)

Figure S5 **Transduction of phosphomimetic moesin enhances S1PR1 internalization.** CD4^+^ T cells from *Msn* ^+/Y^ and *Msn* ^−/Y^ mice were transduced with a control vector or a vector encoding phosphomimetic T558D moesin. Transduced cells were identified by GFP expression. Transduced cells were stimulated with or without 100 nM S1P for 1 h, and processed for quantification of S1PR1 internalization. **, *P*<0.01; ***, *P*<0.001; n.s., not significant (Fisher's exact test).
(TIF)

Figure S6 **Ezrin localization in moesin-deficient CD4^+^ T cells.** (**A**) Localization of S1PR1 and ezrin in moesin-deficient CD4^+^ T cells. Lymph node CD4^+^ T cells from *Msn* ^−/Y^ mice were incubated with or without 10 nM S1P for 10 min, fixed, permeabilized, and stained with anti-S1PR1 and anti-ezrin. Representative confocal images are shown. Scale bar, 5 µm. (**B**) Quantification of cells showing colocalization of S1PR1 and ezrin. The percentages of cells with colocalized or non-colocalized S1PR1 and ezrin at cap-like structures were determined. *n*>60 cells for each group. n.s., not significant (Fisher's exact test).
(TIF)

Movie S1 **Animated volume view depicting colocalization of S1PR1 and Rabaptin-5.** The reconstructed 3D volume corresponding to Figure 4B is rotated around the X-axis.
(AVI)
