# Supplementary figures and images for: Moesin Controls Clathrin-Mediated S1PR1 Internalization in T Cells
Source: PLoS One. 2013 Dec 16;8(12):e82590. doi: 10.1371/journal.pone.0082590 (PMC3865155; doi:10.1371/journal.pone.0082590)

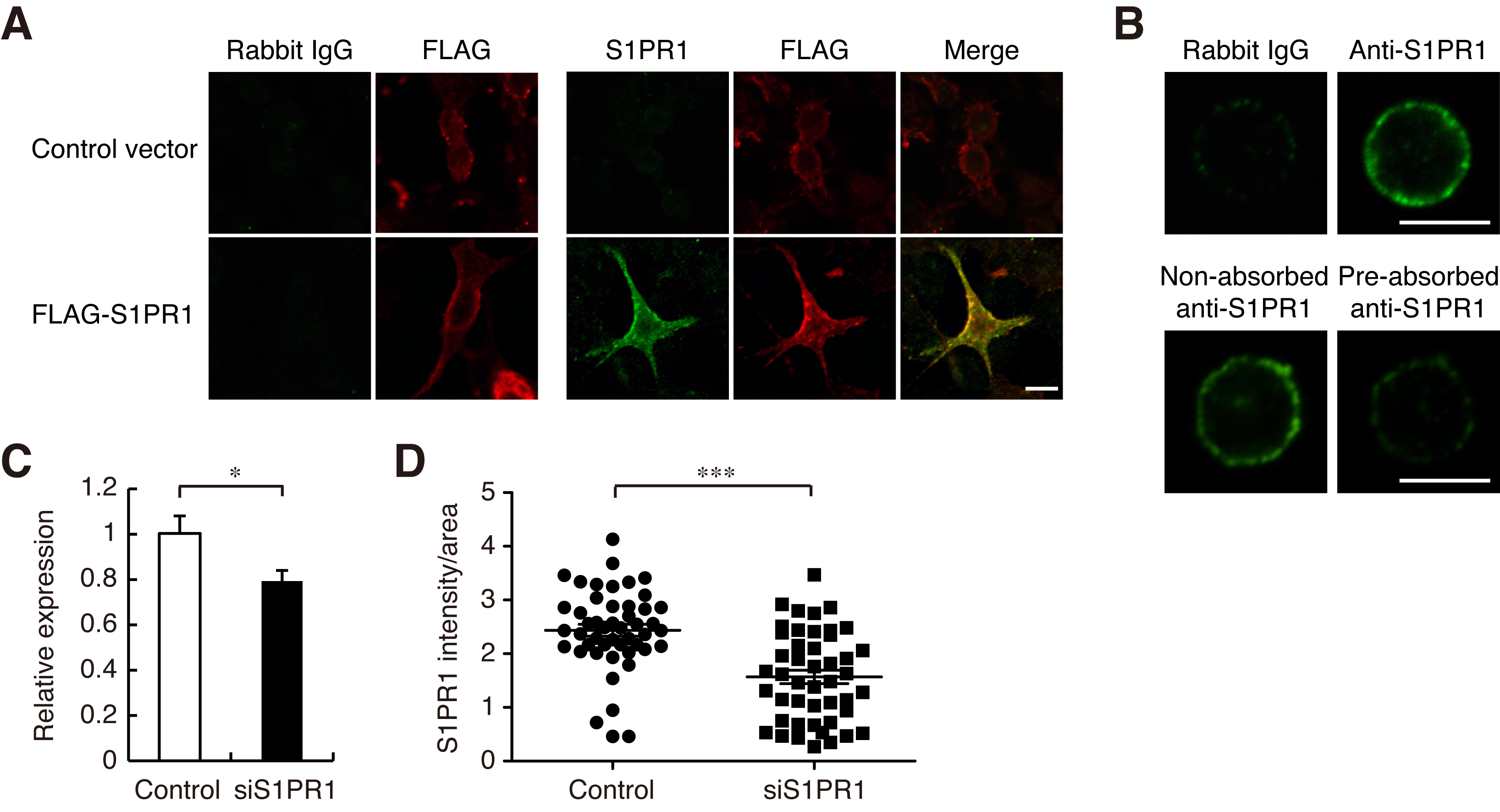

Supplement: Figure S1 — Characterization of the anti-S1PR1 antibody used in this study. (A) Staining of HEK-293 cells with the anti-S1PR1 antibody. Full-length mouse S1PR1 cDNA, amplified from splenic CD4+ T cell cDNA, was inserted into a pFLAG-CMV4 vector (Sigma) between the HindIII and XbaI sites. HEK-293 cells were transfected with a pFLAG-CMV4 vector encoding mouse S1PR1 (FLAG-S1PR1) or a control vector. The transfected cells were fixed, permeabilized, and stained with the anti-S1PR1 (5 µg/ml; ab11424) or control rabbit IgG followed by Alexa Fluor 488-conjugated anti-rabbit IgG. The cells were costained with anti-FLAG (M2; Sigma) followed by Alexa Fluor 594-conjugated anti-mouse IgG. The anti-S1PR1 antibody stained only cells transfected with FLAG-S1PR1. Representative confocal images are shown. Scale bar, 20 µm. (B) Staining of CD4+ T cells with the anti-S1PR1 antibody. Mouse CD4+ T cells were fixed, permeabilized, and stained with the anti-S1PR1 (5 µg/ml; ab11424) or control rabbit IgG followed by Alexa Fluor 488-conjugated anti-rabbit IgG. The cells were also stained with the anti-S1PR1 pre-absorbed with the antigen peptide (ab39763; Abcam) at a molar ratio of 1∶20 overnight. Representative confocal images are shown. Scale bars, 5 µm. (C and D) S1PR1 mRNA expression (C) and quantification of staining with the anti-S1PR1 antibody (D) after partial suppression of S1PR1 in CD4+ T cells. Mouse CD4+ T cells were transfected with siRNA for S1PR1 (Invitrogen) with Neon Transfection System (Invitrogen). After 72 h, total RNA was isolated and subjected to quantitative RT-PCR for S1PR1. Transfected cells were stained with the anti-S1PR1 (5 µg/ml; ab11424) and quantification of fluorescence intensity and area in each cell was performed using ImageJ software (http://rsbweb.nih.gov/ij). ***, P<0.001; *, P<0.05 (Student's t test). (TIF) [file pone.0082590.s001.tif]

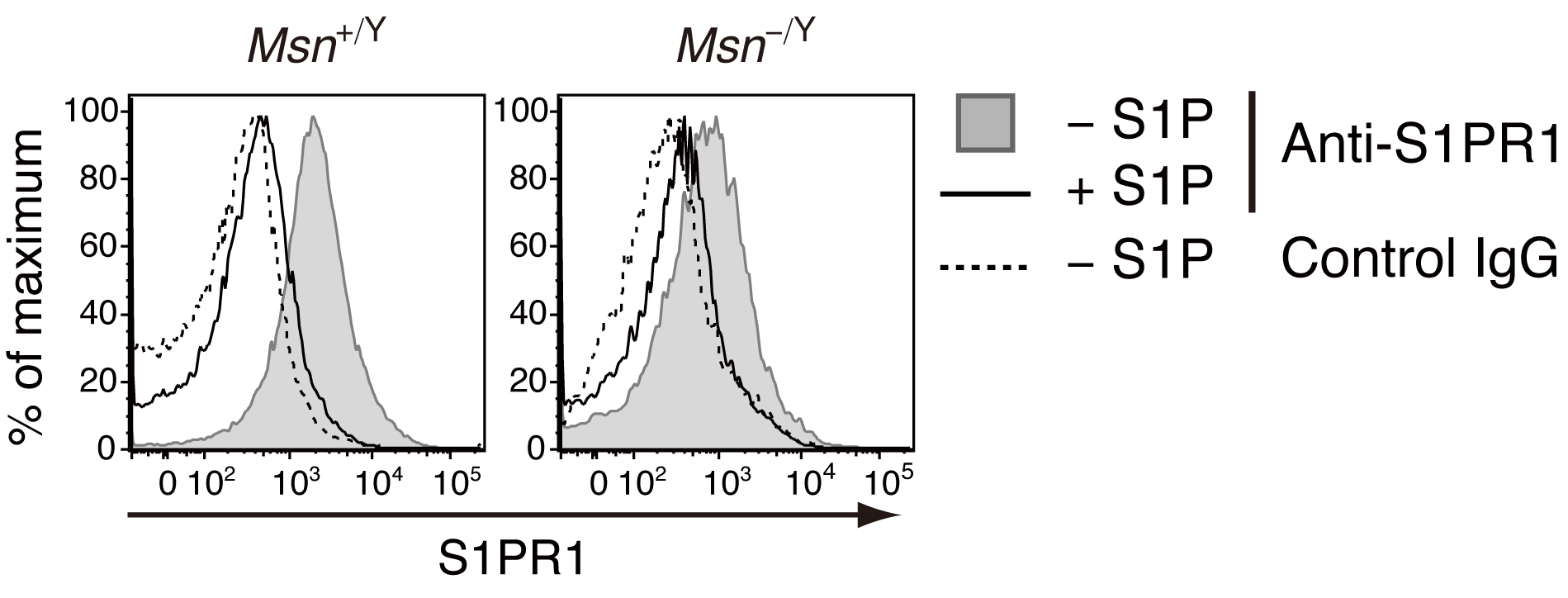

Supplement: Figure S2 — S1P induces cell-surface S1PR1 down-regulation in CD4+ T cells. Lymphocytes from Msn +/Y and Msn −/Y mice were stimulated with or without 100 nM S1P for 1 h. The cells were stained with a rat monoclonal antibody to mouse S1PR1 (40 µg/ml; MAB7089; R&D Systems) at 40 µg/ml or control rat IgG2a for 30 min at room temperature, and then stained with biotinylated anti-rat IgG (Jackson ImmunoResearch, West Grove, PA) followed by allophycocyanin-conjugated streptavidin (BD Biosciences). Lastly, the cells were surface-stained to enable identification of CD4+ T cell subsets. Data were acquired on a LSRFortessa (BD Biosciences) and analyzed using FlowJo. Rat IgG2a staining of S1P-stimulated cells (not shown) largely overlapped to that of unstimulated cells. (TIF) [file pone.0082590.s002.tif]

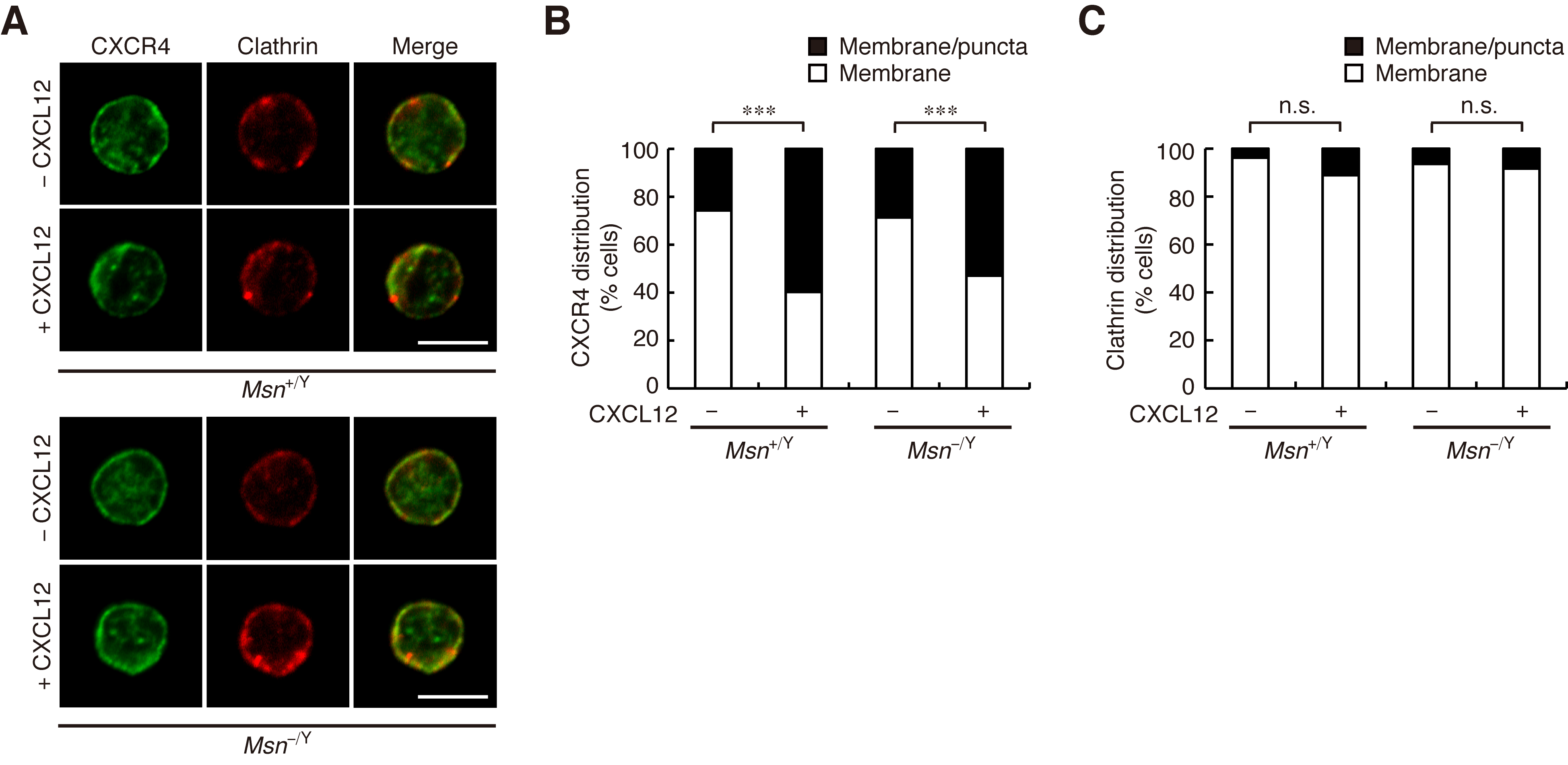

Supplement: Figure S3 — CXCL12-induced CXCR4 localization in CD4+ T cells. (A) CXCR4 and clathrin localization in CD4+ T cells. Lymph node CD4+ T cells from Msn +/Y and Msn −/Y mice were incubated with or without 100 nM CXCL12 for 1 h, fixed, permeabilized, and stained with anti-CXCR4 and anti-clathrin heavy chain. Representative confocal images are shown. Scale bars, 5 µm. (B) Quantification of CXCR4 internalization. Percentages of cells with the indicated distribution patterns of CXCR4 were determined. (C) Quantification of clathrin distribution. The percentages of cells showing the indicated distribution pattern of clathrin were determined. (B and C) n>50 cells for each group. ***, P<0.001; n.s., not significant (Fisher's exact test). (TIF) [file pone.0082590.s003.tif]

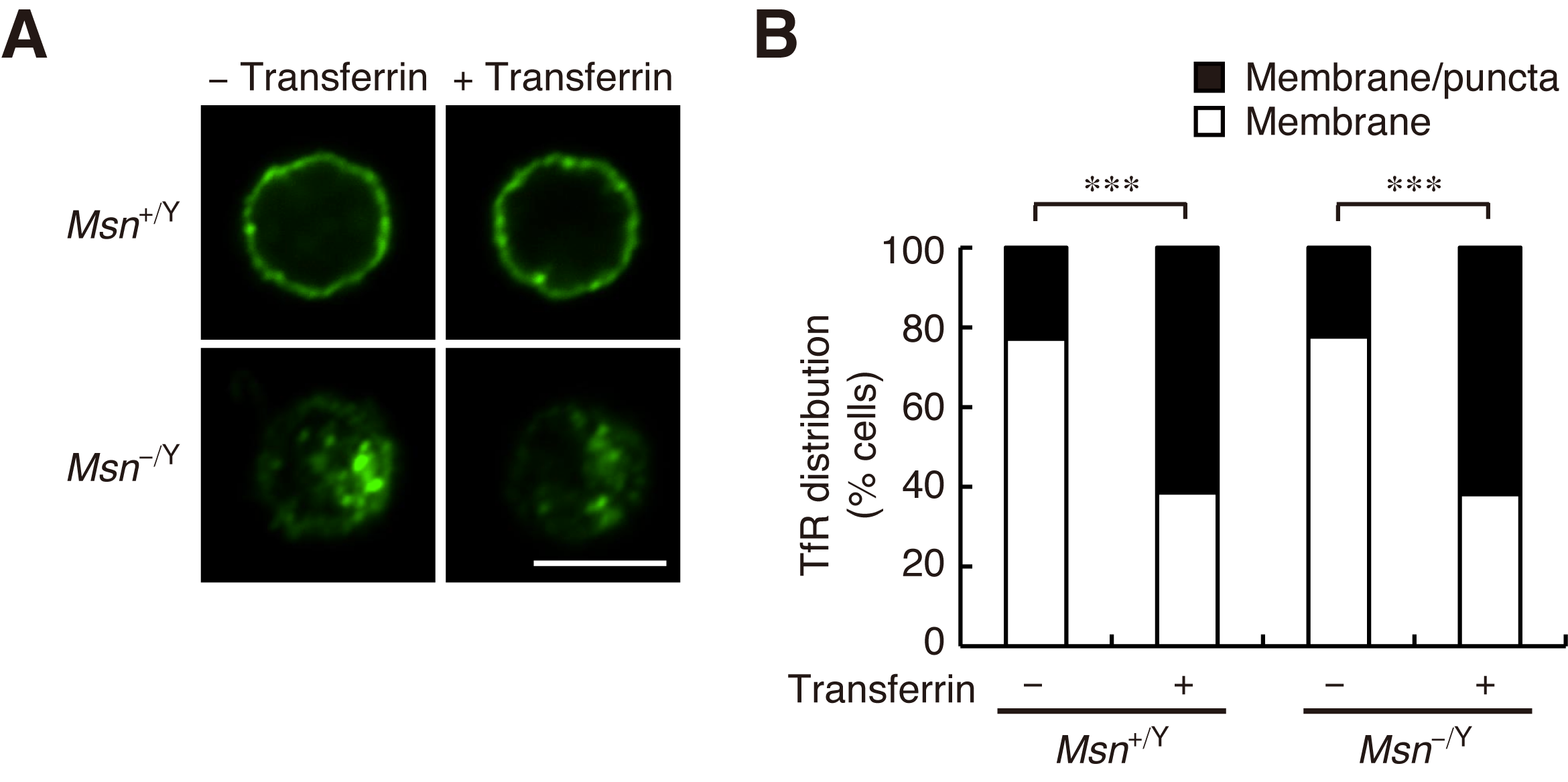

Supplement: Figure S4 — Transferrin-induced transferrin receptor internalization in CD4+ T cells. (A) Transferrin receptor (TfR) localization in CD4+ T cells. CD4+ T cells from Msn +/Y and Msn −/Y mice were incubated with or without 20 µg/ml transferrin (Invitrogen) for 10 min, fixed, permeabilized, and stained with an anti-TfR antibody (Abcam). Representative confocal images are shown. Scale bars, 5 µm. (B) Quantification of TfR internalization. Percentages of cells with the indicated distribution patterns of TfR were determined. n>50 cells for each group. ***, P<0.001 (Fisher's exact test). (TIF) [file pone.0082590.s004.tif]

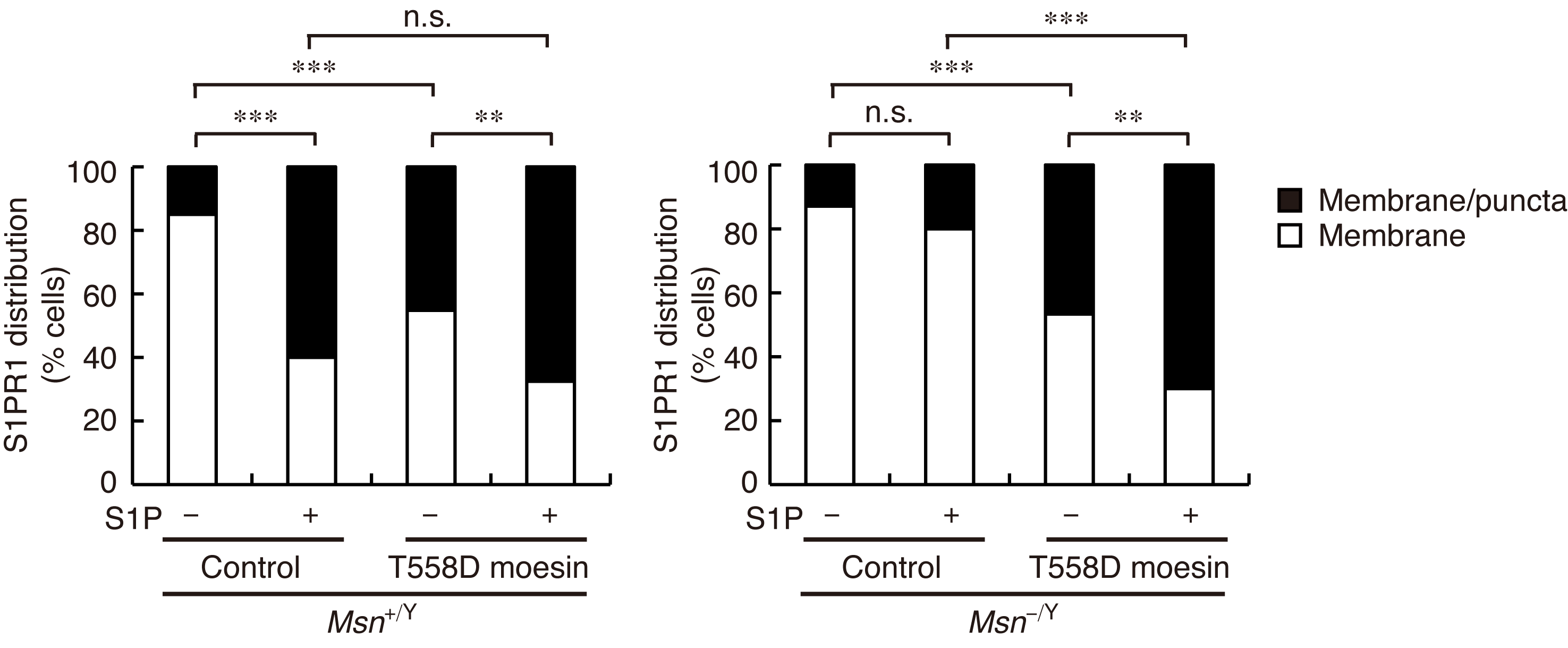

Supplement: Figure S5 — Transduction of phosphomimetic moesin enhances S1PR1 internalization. CD4+ T cells from Msn +/Y and Msn −/Y mice were transduced with a control vector or a vector encoding phosphomimetic T558D moesin. Transduced cells were identified by GFP expression. Transduced cells were stimulated with or without 100 nM S1P for 1 h, and processed for quantification of S1PR1 internalization. **, P<0.01; ***, P<0.001; n.s., not significant (Fisher's exact test). (TIF) [file pone.0082590.s005.tif]

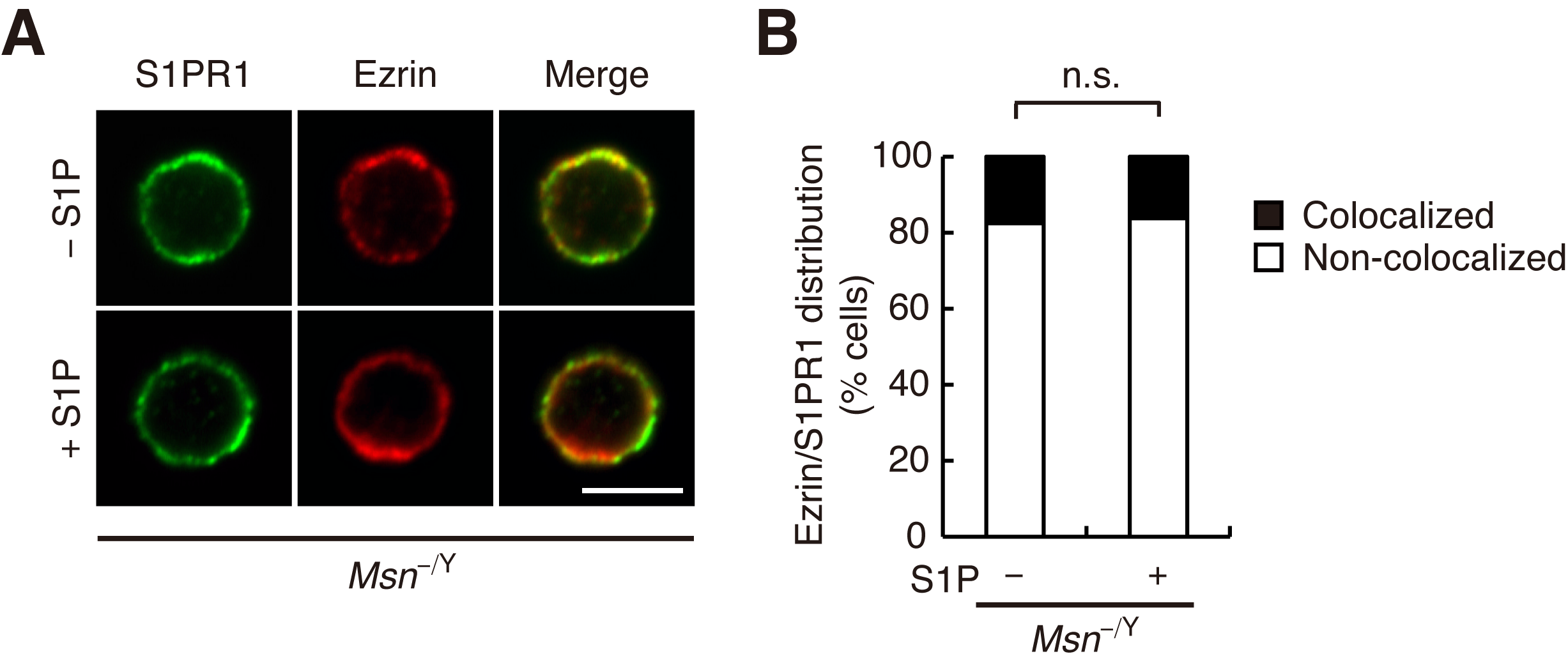

Supplement: Figure S6 — Ezrin localization in moesin-deficient CD4+ T cells. (A) Localization of S1PR1 and ezrin in moesin-deficient CD4+ T cells. Lymph node CD4+ T cells from Msn −/Y mice were incubated with or without 10 nM S1P for 10 min, fixed, permeabilized, and stained with anti-S1PR1 and anti-ezrin. Representative confocal images are shown. Scale bar, 5 µm. (B) Quantification of cells showing colocalization of S1PR1 and ezrin. The percentages of cells with colocalized or non-colocalized S1PR1 and ezrin at cap-like structures were determined. n>60 cells for each group. n.s., not significant (Fisher's exact test). (TIF) [file pone.0082590.s006.tif]
